# Immuno-inflammatory pathogenesis in ischemic heart disease: perception and knowledge for neutrophil recruitment

**DOI:** 10.3389/fimmu.2024.1411301

**Published:** 2024-07-10

**Authors:** Yumeng Wang, Xintian Shou, Yang Wu, Dong Li

**Affiliations:** ^1^ Department of Traditional Chinese Medicine, Beijing Tsinghua Changgung Hospital, School of Clinical Medicine, Tsinghua University, Beijing, China; ^2^ Cardiovascular Diseases Center, Xiyuan Hospital, China Academy of Chinese Medical Sciences, Beijing, China; ^3^ Department of Cardiovascular, Dongfang Hospital, Beijing University of Chinese Medicine, Beijing, China

**Keywords:** neutrophil recruitment, immunoinflammation, ischemic heart disease, aseptic inflammation, innate immunity

## Abstract

Ischemic heart disease (IHD) can trigger responses from the innate immune system, provoke aseptic inflammatory processes, and result in the recruitment and accumulation of neutrophils. Excessive recruitment of neutrophils is a potential driver of persistent cardiac inflammation. Once recruited, neutrophils are capable of secreting a plethora of inflammatory and chemotactic agents that intensify the inflammatory cascade. Additionally, neutrophils may obstruct microvasculature within the inflamed region, further augmenting myocardial injury in the context of IHD. Immune-related molecules mediate the recruitment process of neutrophils, such as immune receptors and ligands, immune active molecules, and immunocytes. Non-immune-related molecular pathways represented by pro-resolving lipid mediators are also involved in the regulation of NR. Finally, we discuss novel regulating strategies, including targeted intervention, agents, and phytochemical strategies. This review describes in as much detail as possible the upstream molecular mechanism and external intervention strategies for regulating NR, which represents a promising therapeutic avenue for IHD.

## 1 Introduction

Neutrophils, representing the predominant fraction of granulocytes in the majority of mammalian species, serve an integral function within the innate immune defense mechanism. They are short-lived, nonspecific, and highly mobile, enabling entry into the tissue where other cells/molecules cannot invade. Aseptic inflammation is the main response of innate immunity and the necessary condition to trigger and drive vascular diseases. After the onset of aseptic inflammation, neutrophils are the first leukocytes to be recruited to the site of inflammation and subsequently promote the immune response (1). A moderate immune response coordinates a beneficial dynamic homeostatic response and contributes to tissue repair, while an exaggerated response can cause additional damage. Ischemic heart diseases (IHD), such as myocardial infarction (MI), myocardial ischemia-reperfusion(I/R) injury, and heart failure (HF), can initiate innate immune responses, induce aseptic inflammation (2), and culminate in the mobilization and proliferation of neutrophils (3).

Neutrophil recruitment (NR) is pivotal in mediating the aseptic inflammatory response associated with IHD (3). Pathological recruitment of neutrophils is triggered by changes in the surface of endothelial cells caused by inflammatory mediator (including histamine, cysteinyl leukotrienes, and cytokines) stimulation (4–7). Once recruited, neutrophils can release various inflammatory and chemotactic mediators that cause cell damage and in turn further facilitate the recruitment process; it can also block small blood vessels at the site of inflammation, thereby impeding blood flow (8, 9). Animal studies and clinical studies have demonstrated that increased neutrophil counts correlate with the severity of coronary artery damage in patients with coronary artery disease (CAD) (10, 11). Neutrophil-to-leukocyte ratio (NLR) has been shown to be an independent predictor of outcome in patients with stable CAD, as well as a predictor of short- and long-term mortality in patients with acute coronary syndrome (ACS), ST-segment elevation myocardial infarction (STEMI), and heart transplantation (12–14). Consequently, the recruitment and subsequent accumulation of neutrophils may serve as key determinants of sustained cardiac inflammation in the context of IHD. Therapeutic modulation and inhibition of these processes may prove beneficial in attenuating myocardial inflammation and impeding the advancement of IHD pathology.

Recent studies have predominantly directed attention toward elucidating the global contribution of neutrophils to ischemic heart disease pathophysiology. In contrast, the mechanisms governing neutrophil recruitment have been comparatively underexplored, largely attributable to the pronounced heterogeneity in initiating signals for neutrophil recruitment across diverse tissue (15, 16). In recent years, the upstream molecular mechanism and external intervention strategies for regulating NR have attracted the attention of many scholars, and many satisfactory results have been obtained. However, the findings have not been systematically summarized. This paper intends to summarize the specific process and influence of NR in IHD, as well as the molecular mechanism of regulating NR and effective intervention measures, in order to provide ideas for the study of ischemic heart disease from this perspective.

## 2 Cascade reactions of NR in IHD

NR is considered to be a series of cascade reactions: tethering, rolling, adhesion, crawling, and transmigration. When cardiac ischemia occurs, aseptic inflammation induces inflammatory mediators to stimulate endothelial cells. P-selectin and E-selectin bind to their glycosylated ligands, allowing neutrophils to be tethered and trapped in fast-flowing blood and subsequently roll with the flow along the vascular endothelium. Chemokines activate neutrophils by binding to their receptors. Then, neutrophils express integrins (LFA1 and MAC1) binding to endothelial surface adhesion molecule (ICAM-1/ICAM-2), promoting stable adhesion of neutrophils to vascular endothelial cells. Finally, neutrophils crawl at the endothelial cell-cell junction, release protease to digest the endothelial basement membrane (EBM), and complete vertical transmigration through paracellular (between endothelial cells) and transcellular (through endothelial cells). The process of transmigration also requires the involvement of integrins, CAM, and multiple connexins.

## 3 Role of NR in IHD

### 3.1 Atherosclerosis

**Figure 1:**
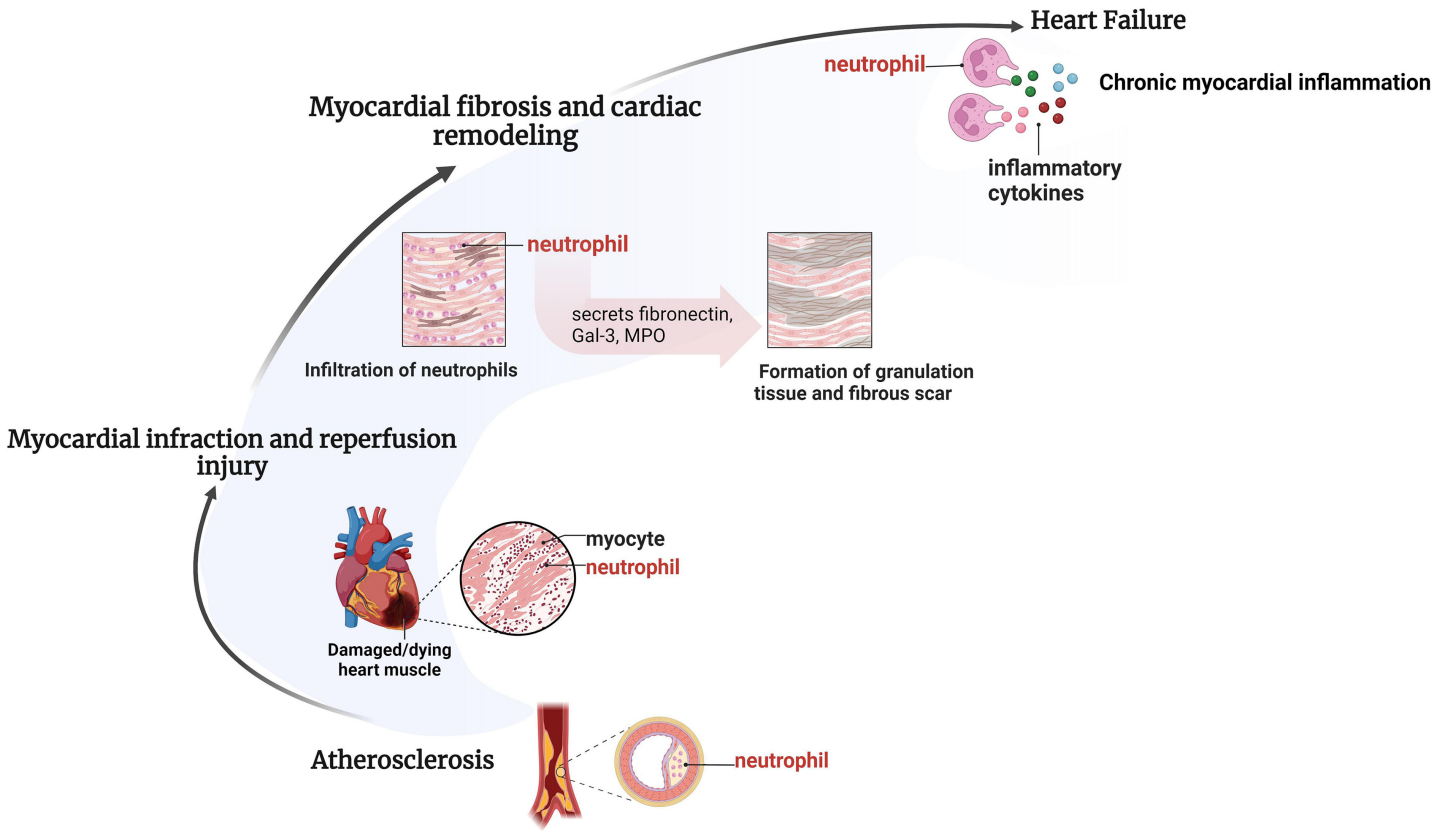
Pathological processes of different IHD involving neutrophils. Neutrophil recruitment takes place in various pathological processes of different IHD. Neutrophil recruitment enhances atherosclerotic plaque formation, exacerbates inflammatory responses and myocardial injury during myocardial infarction and reperfusion injury, speeds up myocardial fibrosis and ventricular remodeling, resulting in persistent chronic inflammation and facilitating the onset of late-stage heart failure. Created by BioRender.

Neutrophils are the main cell group interacting with atherosclerotic endothelium, as well as the main cell component of atherosclerotic lesions. Clinical and experimental investigations have consistently demonstrated a marked presence of neutrophils within aortic plaques, particularly concentrated in the shoulder regions where inflammatory activity is notably heightened (17, 18). NR plays an important role in atherosclerosis and plaque rupture. First, neutrophils recruited in the plaque will release granular protein, which will be deposited in endothelial cells and vascular walls to adhere to monocytes, activate macrophages, release proinflammatory factors, and thus aggravate the inflammatory response. Secondly, the proteolytic enzyme released by granular protein can weaken the fibrous cap and promote plaque rupture. Finally, myeloperase (MPO) released by granular protein induces vascular endothelial cell apoptosis by reducing the utilization of NO, leading to plaque erosion (19). In addition, cholesterol crystals in plaques also trigger neutrophils to release neutrophil extracellular traps (NETs), which indirectly promote NR in atherosclerotic plaques by promoting the release of macrophages and activating T-helper 17 (TH17) cells (**Figure 1**).

### 3.2 Myocardial infarction

Myocardial infarction can quickly activate the innate immune pathway, and trigger a strong inflammatory response in the early stage. The necrosis of myocardial cells and stroma will promote the occurrence of damage related molecule pattern (DAMP) (20), which can be recognized by toll-like receptor (TLR), and promote the activation of innate immune cells and inflammation (21). The inflammatory response caused a marked upregulation of chemokines in the infarcted myocardium, which facilitated the onset of the NR. Meanwhile, ROS mediated by myocardial ischemia can also promote NR. The process of neutrophil recruitment changed with time after myocardial infarction. Histological examination showed that neutrophils appeared 12–24 hours after the ischemic attack and reached the peak on the third day, which suggested that neutrophils were recruited most in the early stage (1–3 days) after myocardial infarction and were mainly N1 polarized proinflammatory neutrophils. From the 5th to the 7th day, the proportion of N2 polarized anti-inflammatory neutrophils increased (22, 23). After 7 days, neutrophils began to disappear from the infarcted area, and lymphocytes and monocytes gradually increased (24). It should be noted that the transmigration of neutrophils through the endothelium, the core step of NR, occurs in both ischemic and border regions.

### 3.3 Myocardial ischemia-reperfusion injury

Neutrophils are the main type of inflammatory cells in myocardial I/R injury. The pathological mechanism of NR in myocardial I/R injury is consistent with that of myocardial infarction, as both involve aseptic inflammatory reactions triggered by the activation of innate immune pathways. Myocardial ischemia mainly affects the myocardial tissue in the central necrotic area of the occluded coronary artery perfusion area. The process of reperfusion limits the infarct size of the central necrotic area but starts molecular events that lead to lethal reperfusion injury in the border area (25). After permanent coronary artery occlusion, neutrophil infiltration is mainly limited to the edge of the infarcted area. The process of reperfusion changes the distribution of neutrophils, making them recruit in the infarcted area of the myocardium (24). Neutrophils infiltrate into the infarcted area, producing a large number of reactive oxygen species (ROS) and proteolytic enzymes (26), aggravating the damage of myocardial tissue, which in turn maximally recruited and activated more neutrophils, forming a vicious circle.

### 3.4 Heart failure

Acute inflammation is an essential mechanism for heart repair and defense after injury. Systemic inflammation has been recognized as a pathobiological feature of acute and chronic heart failure, associated with the development, progression, and complications of HF, and predicted adverse outcomes independently of other clinical parameters such as left ventricular ejection fraction (27). The timely clearance of neutrophils is the first critical step in the regression of inflammation during the healing process after MI-induced myocardial injury. The production of locally endogenous peptides/bioactive lipids by neutrophil terminates NR, which is a marker of inflammation resolution. However, if neutrophils are not cleared on time and remain in a state of continuous recruitment, chronic inflammation and advanced heart failure will occur (28). The innate immune system is active in patients with chronic heart failure, accompanied by a persistent chronic inflammatory response. Neutrophils recruited into the myocardium release more pro-inflammatory factors than anti-inflammatory factors, which can further exacerbate myocardial damage and cardiac decline. It has been shown that higher neutrophil levels in the first 12 hours after acute myocardial infarction predict the development of chronic HF (29).

### 3.5 Myocardial fibrosis and cardiac remodeling

Inflammation plays a key role in poor ventricular remodeling and decreased cardiac function after injury (30). Overactive inflammatory signaling can aggravate myocardial damage and lead to poor cardiac remodeling. The N2-type neutrophils express anti-inflammatory molecules in the late stage of cardiac remodeling, and their proportion increases continuously after myocardial infarction, reaching nearly 20% on the 7th day (23). On day 3 of myocardial infarction, neutrophils initiate apoptosis, reduce inflammatory signaling, and begin to help ECM reorganize. Neutrophils recruited to the infarct area remove dead cells and matrix debris by phagocytosis in preparation for scarring in the area. On days 5 to 7 of myocardial infarction, neutrophils produce ECM proteins needed for scarring, including fibronectin, Gal-3, and fibrinogen (31). In addition, the heme MPO released by the primary granules of neutrophils can promote the deposition of myocardial collagen in the atrium and ventricle, which is the key medium for myocardial remodeling. Recruitment of neutrophils leads to increased concentrations of MPO, and the oxidant produced by MPO can lead to impaired myocardial function and poor ventricular remodeling after myocardial infarction (32, 33). Therefore, over recruitment of neutrophils leads to excessive myocardial collagen deposition and ECM remodeling, leading to the formation of mature scars, promoting myocardial fibrosis, and compromising organ compliance (**Figure 1**).

## 4 Molecular mechanisms regulating NR in IHD

### 4.1 Immune-related molecular mechanisms

#### 4.1.1 Immune receptors and ligands

**Figure 2:**
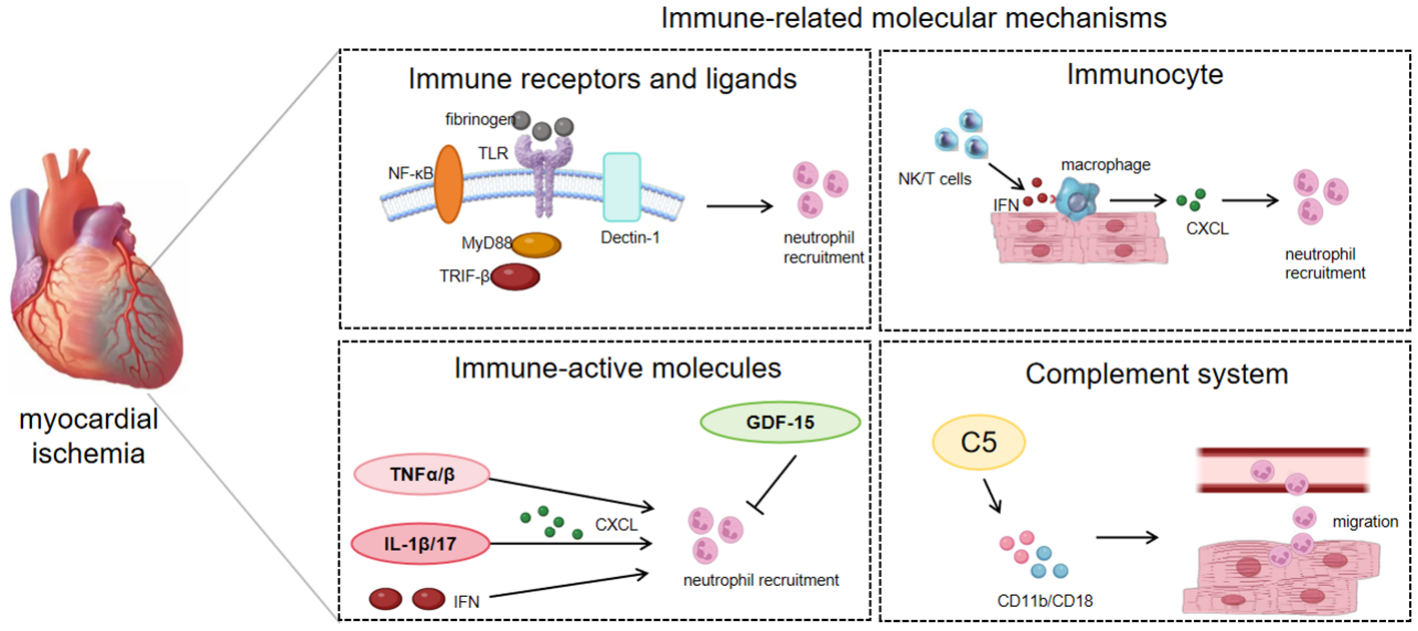
Immune-related regulatory mediators of NR in IHD pathophysiology.

Immune receptors and their corresponding ligands are instrumental in mediating the homing of neutrophils to ischemic myocardial tissue. For example, transmembrane protein receptor dectin-1 can promote the expression of chemokine CXCL1 and granulocyte colony-stimulating factor (G-CSF) in macrophages through the spleen tyrosine kinase (Syk)/nuclear factor kappa-B(NF-κB) signaling pathway, and can also regulate the production of interleukin (IL) 23 and IL-1β, affecting neutrophil recruitment and myocardial I/R injury (34). The upregulation of NF-κB p65 expression in ECs increases the expression of adhesion molecules, leading to robust infiltration of neutrophils and disorganization/degradation of ECM in the myocardial ischemic border zone (35). TLR can affect signal transduction of its downstream protein myeloid differentiation primary response gene 88(MyD88) or TIR domain-containing adaptor inducing interferon (TRIF) -β, initiating neutrophil recruitment in the I/R myocardium (36). In addition, in animal models of heart failure, fibrinogen (FN) can act as a ligand for TLR2 and TLR4, promote the process of neutrophil adhesion by regulating TLR and cardiac endothelial cell adhesion molecules, and participate in neutrophil recruitment (30) (**Table 1**; **Figure 2**).

**Table 1:** Immune-related regulatory mediators of NR in IHD pathophysiology.

	Key Mediators	Effect on NR	Function	IHD diseases	Ref.
Immune receptors	Dectin-1	Promote	Promote CXCL1 and G-CSF through Syk/NF-κB signaling pathway, regulate the production of IL-23 and IL-1β	Myocardial I/R injury	(34)
	TLR	Promote	Affect MyD88 or TRIF-β	Myocardial I/R injury	(36)
	NF-κB p65	Promote	Increase the expression of adhesion molecules	MI, Myocardial I/R injury	(35)
Immune ligands	fibrinogen	Promote	As a ligand for TLR-2 and TLR-4, promote neutrophil adhesion	HF	(30)
Immune-active molecule	GDF-15	Inhibit	Through the ALK-5/TGF-βRII heterodimer	MI	(37, 38)
	IL-1β, IL-17	Promote	Drives the chemokines CXCL1 and CXCL2	AS	(39–41)
	TNFα, TNFβ	Promote	Contributes to the development of contractile dysfunction	IR, HF	(42)
	Type I interferons	Promote	Rely on the TLR4/TRIF pathway to promote neutrophil adhesion	Myocardial I/R injury	(36)
Immunocyte	macrophage	Promote	Facilitate the transendothelial migration through TLR9/MyD88/CXCL5 signaling	Myocardial I/R injury	(43)
	mast cell	Promote	Cause vascular fluid leakage and edema	Myocardial I/R injury	(44)
	Natural killer (NK) cells and T lymphocytes	Promote	Produce inflammatory mediators to stimulate resident macrophages to attract neutrophils	MI	(45)
	Platelets	Promote	Promote neutrophils activation and adhesion	Myocardial I/R injury	(46)
Complement System	C5	Promote	Upregulate CD11b/CD18 and cause transendothelial migration	HF	(47)

ALK, anaplastic lymphoma kinase; G-CSF, granulocyte colony-stimulating factor; GDF, growth differentiation factor; IL,interleukin; I/R, ischemia reperfusion; MI, myocardial infarction; MyD88, myeloid differentiation primary response gene 88; NF-κB, nuclear factor kappa-B;TGF, transforming growth factor; TLR, toll-like receptor; TRIF, TIR domain-containing adaptor inducing interferon; Syk, spleen tyrosine kinase.

#### 4.1.2 Immune-active molecule

Many immune-active molecules, encompassing a diverse array of cytokines, exert influence over the process of neutrophil recruitment in the context of IHD. Growth differentiation factor (GDF)-15, a transforming growth factor beta (TGF-β)-related cytokine, is an inhibitor of leukocyte integrin activation required for survival after myocardial infarction in mice and inhibits NR process in mice through anaplastic lymphoma kinase (ALK)-5/TGF-βRII heterodimer (37, 38). IL-1β, secreted by macrophages, upregulates the T-cell-derived cytokine IL-17, which drives the chemokines CXCL1 and CXCL2 to promote NR and amplify the inflammatory response (39). Tumor necrosis factor (TNF) α and TNFβ also enhance the activation and recruitment of neutrophils in the myocardium, thereby further damaging the myocardium (42). Type I interferons can rely on the TLR4/TRIF pathway to promote neutrophil adhesion to coronary endothelial cells, thus coordinating neutrophil recruitment to injured myocytes (36).

#### 4.1.3 Immunocyte

Various subtypes of macrophages are primarily responsible for encouraging the recruitment of neutrophils in IHD. The cytokine interleukin-1β (IL-1β) secreted by macrophages can up-regulate the T-cell-derived cytokine IL-17, which drives the chemokines CXCL1 and CXCL2 to promote neutrophil recruitment and amplify inflammation (43). Heart-resident CCR2^+^ macrophages facilitate the transendothelial migration of neutrophils into injured hearts through TLR9/MyD88/CXCL5 signaling (43). In addition, the process of degranulation and mediator release of mast cells contributes to the inflammatory response, causing vascular fluid leakage and edema, and promoting NR (44). Natural killer (NK) cells and T lymphocytes produce inflammatory mediators such as IFN-γ, which stimulate resident macrophages to attract neutrophils to cardiac tissue (45). Platelets can also aggravate myocardial I/R damage by promoting neutrophil activation and adhesion (46).

#### 4.1.4 Complement system

When myocardial ischemia occurs, inflammation stimulates complement activation and accelerates neutrophil adherence. The strong neutrophil attraction of C5a causes neutrophils to adhere firmly to endothelial cells by upregulating CD11b/CD18 (Mac-1) and causing transendothelial migration (48). In addition, C5a also stimulates neutrophils to produce superoxide, which aggravates the occurrence of oxidative stress (49). Therefore, inhibition of C5 may reduce neutrophil infiltration during myocardial I/R injury in a manner that provides cardiac protection (47) (**Table 1**; **Figure 2**).

### 4.2 Non-immune-related molecular mechanisms

Numerous pro-resolving lipid mediators that are produced by neutrophils in turn affect their own recruitment. For example, lipid toxin A4, a dual anti-inflammatory and pro-resolution lipid mediator, induces changes in the phosphorylation of cytoskeletal proteins, leading to inhibition of neutrophil migration (50, 51). The protectins can also play a similar role in inhibiting neutrophil infiltration (52). Annexins are a widely distributed class of calcium-dependent phospholipid binding proteins. Annexin 1 inhibits neutrophil adhesion and migration in a mitogen-activated protein kinase (MAPK)-dependent manner via formyl peptide receptor (FPR) signaling (53–55), and its mimics exhibit the same cardioprotective effect through modulation of immune cell activation in myocardial infarction (56).

Various proteins released in physiological or pathological states affect the recruitment process of neutrophils and become biomarkers of inflammatory response in IHD. Angiopoietin-2 (Angpt2), recognized as a biomarker of poor outcome in IHD, plays a critical role in MI. In the acute phase of MI, endothelial-derived Angpt2 antagonized Angpt1/Tie2 signaling, which was greatly involved in increased adhesion molecular expression, enhanced neutrophil infiltration, and intensified inflammatory response (35). S100A8∕A9, a kind of Ca^2+^ binding protein, has become a valuable biomarker and treatment target to detect and modulate neutrophil involvement in myocardial infarction. Studies have shown that its blockers can significantly reduce neutrophil infiltration in infarcted hearts (57). Developmental endothelial site-1 (DEL-1) is an anti-inflammatory glycoprotein whose deficiency exacerbates pressure overload-induced heart failure by promoting neutrophil infiltration and the formation of neutrophil extracellular traps (58). transcriptional co-activator yes-associated protein (YAP) reduced cardiomyocyte (CM) necrosis and neutrophil infiltration after I/R stress (59).

Some biological processes can also affect NR processes in IHD. Adipose tissue lipolysis and cardiomyocyte lipid accumulation augmented cardiac inflammation. Inhibition of adipose tissue lipolysis reduces lipid droplet accumulation, attenuates neutrophil infiltration, and improves cardiac function (60). Exocytosis is also a factor affecting NR. Studies have shown that neutrophil exocytosis inhibitors (Nexinhib20) not only help reduce the exocytosis process, but also inhibit neutrophil adhesion and activation of β2 integrin, thereby reducing neutrophil recruitment (61). By enhancing M2 macrophage-induced efferocytosis of apoptotic neutrophils, mesenchymal stem cells markedly decrease neutrophil number and improve cardiac repair after myocardial I/R in rats (62). In addition, enhancing vascular integrity and inhibiting the high permeability of cardiac microvascular endothelium (CME) contributed to reducing the expression of vascular cell adhesion molecule 1 (VCAM-1) on CME cells, leading to reduced neutrophil infiltration (63).

## 5 Interventions to regulate NR

### 5.1 Targeted intervention

Targeted inhibition of key mediators within the cascade reactions weakens NR. Both MPO-knockout models (64) and MPO inhibitors (65) lead to reduced neutrophils recruitment, and improved ventricular function and remodeling in myocardial infarction. The absence of NR adhesion molecules also inhibited NR (66). Receptor antagonists of cytokines and chemokines, such as IL-1 receptor antagonist (67) and NLRP3-inflammasome inhibitor (68), reduce the extent of neutrophil infiltration and myocardial infarction size. Targeted regulation of related proteins or genes in the molecular mechanism of NR directly inhibits the NR process. Studies have shown that ferrostatin-1 (Fer-1), as a specific inhibitor of ferroptosis, can reduce the level of pro-ferroptotic hydroperoxy-arachidonoyl-phosphatidylethanolamine, reduce cardiomyocyte apoptosis and block neutrophil recruitment (36).

Targeting adenosine helps to regulate the NR process. Adenosine A2b receptor (Adora2b), as a G-protein-coupled receptor, its activation inhibits neutrophils from releasing TNF-α, which in turn reduces NR and reduces I/R damage (42, 69, 70). The adenosine analogue AMP579 alleviates heart damage by inhibiting neutrophil activation and excessive vascular adhesion (71, 72). Targeted inhibitors of adenosine hydrolase help to inhibit NR. Targeting PDE4B (the hydrolase of cyclic adenosine monophosphate) inhibits neutrophil-endothelial cell interaction and expression of cell adhesion molecules, neutrophil cardiac infiltration, and release of proinflammatory cytokines, exerting cardioprotection in acute myocardial infarction (73).

In ischemic heart disease animal models, targeted depletion of complement significantly leads to a considerable reduction in neutrophil numbers (74). The C3 inhibitor, sCR1, reduces the infarct size and minimizes the accumulation of neutrophils in the infarct area, possibly by reducing C5a production, promoting adhesion receptor expression, and promoting chemotactic processes (75, 76). Anti-C5 therapy in the setting of MI/R significantly inhibits cell apoptosis, necrosis, and neutrophil infiltration (77). The recombinant human C5a receptor antagonist CGS 32359 inhibits surgical I/R injury after coronary occlusion and reduces the extent of high-risk myocardial infarction associated with reduced neutrophil aggregation (78).

### 5.2 Agents intervention

Several clinically used drugs have been found to either directly or indirectly regulate NR (**Table 2**). Metoprolol has been shown to inhibit neutrophil migration in an Adrenergic receptor (ADR) β1-dependent manner (79). During the early stages of neutrophil recruitment, it inhibits the structural and functional changes necessary for the effective engagement of circulating platelets, resulting in erratic intravascular dynamics, weakened neutrophil-platelet interactions, and blunted inflammation. Trimetazidine has been shown to inhibit neutrophil accumulation after myocardial ischemia and reperfusion (80). Empagliflozin prevented the permeation of neutrophils into the myocardium and therefore suppressed the transcription of these inflammatory cytokines (81). Metformin, as a first-line drug for hypoglycemia, can also inhibit NR in IHD. It has been shown to reduce myocardial neutrophil activity and cardiac remodeling after myocardial infarction through the AMPK pathway, thereby alleviating myocardial injury (82). Colchicine has been shown to inhibit neutrophil proliferation through inflammatory signaling pathways and reduce microvascular obstruction after myocardial I/R injury (83).

**Table 2:** Agents interventions to regulate NR.

Drugs	IHD diseases	Molecular mechanisms	Ref.
Metoprolol	MI, myocardial I/R injury	Targeting ADRB1	(79)
Trimetazidine	Myocardial I/R injury	Unknown	(80)
Empagliflozin	Myocardial I/R injury	Activate the AMPKα1/ULK1/FUNDC1/mitophagy pathway	(81)
Metformin	MI	Activatte AMPK pathway	(82)
Colchicine	Myocardial I/R injury	Inhibit S100A8/A9-NLRP3/IL-1β/IL-1R pathway	(83)

ADRB, adrenergic receptor; AMPK, AMP-activated protein kinase; FUNDC, FUN14 domain-containing protein; IL,interleukin; I/R, ischemia reperfusion; MI, myocardial infarction; ULK, Unc-51-like autophagy activating kinase.

### 5.3 Phytochemical strategies

Herbal medicines have been widely utilized alone or as a supplement and alternative therapy to treat various disorders in East Asia because of their reduced toxicity, fewer side effects, and cheaper cost (84). Many herbal ingredients may act synergistically to protect against myocardial injury in IHD through suppressing NR, such as salvianolic acid b, stigmasterol, resveratrol (85), tetrandrine (86), baicalin (87), silibinin (88), 2-methoxycinnamaldehyde (89), celastrol (90), et al. (**Table 3**). These phytochemicals, derived from different herbs, alleviated the inflammatory response of IHD to varying degrees by inhibiting NR through different molecular pathways.

**Table 3:** Phytochemical strategies to regulate NR.

Chemical name	Sdf structure	IHD diseases	Molecular mechanisms	Ref.
Salvianolic acid b	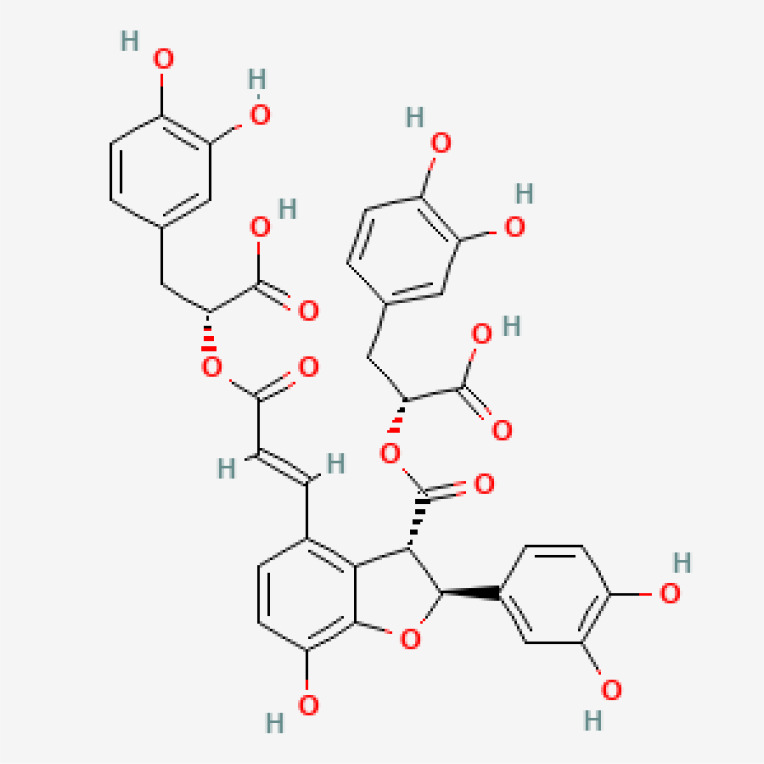	MI	Bingding with TLR4	(85)
Stigmasterol	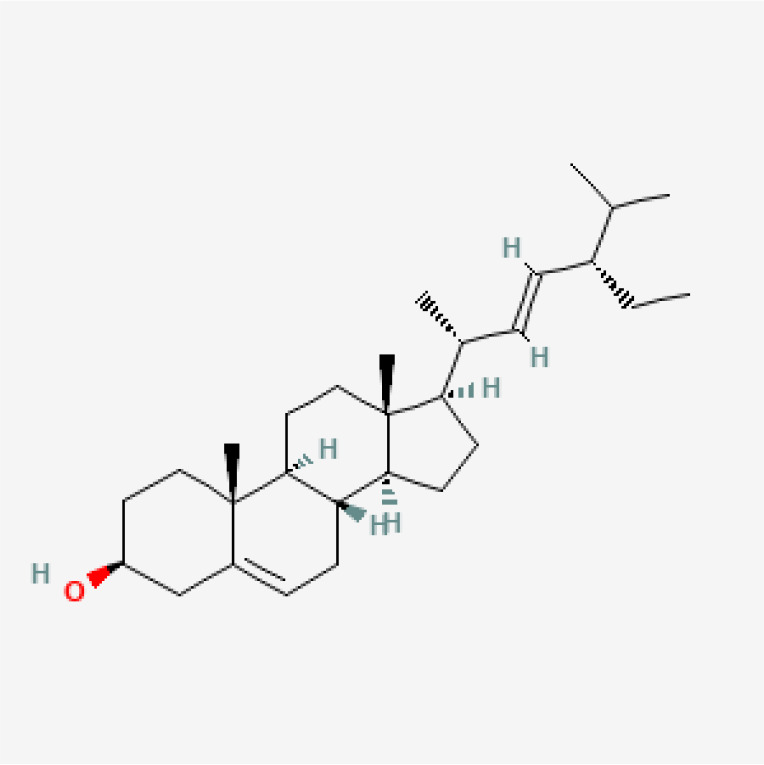	MI	Bingding with TLR4	(85)
Resveratrol	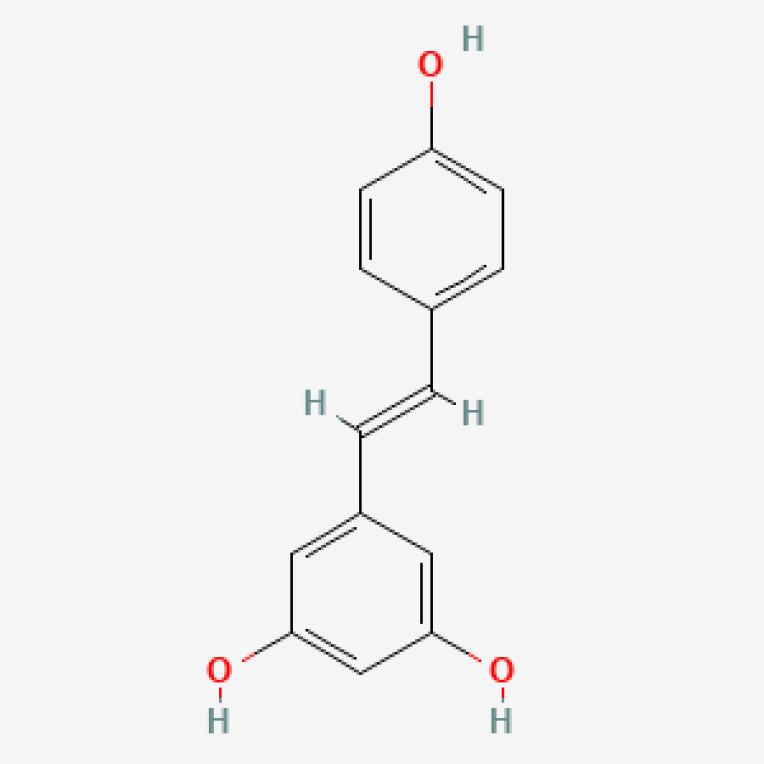	MI	Bingding with TLR4	(85)
Tetrandrine	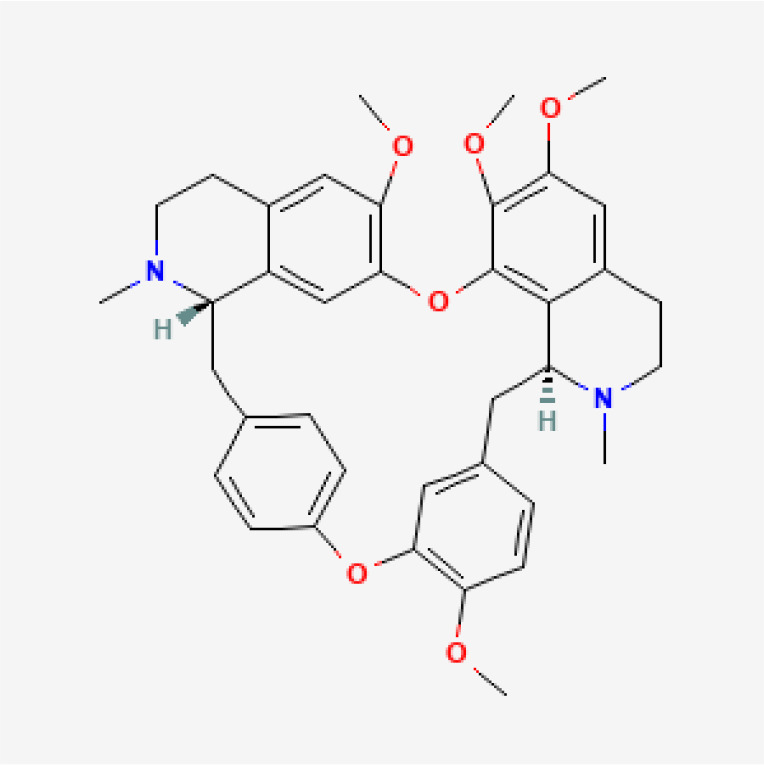	Myocardial I/R injury	Inhibit N-fMLP-induced adhesion and ROS production	(86)
Baicalin	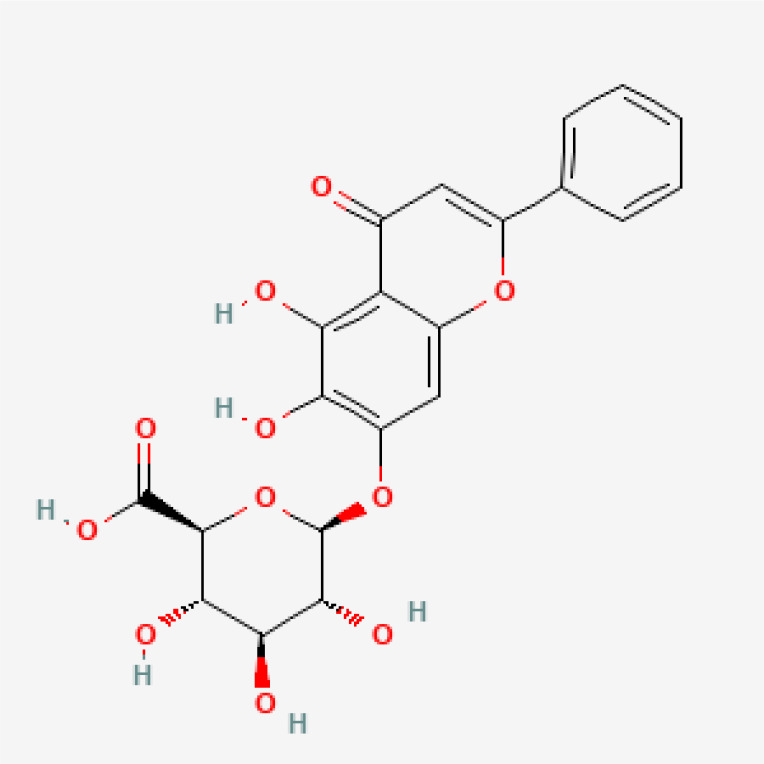	Myocardial I/R injury	Inhibit JAK/STAT pathway	(87)
Silibinin	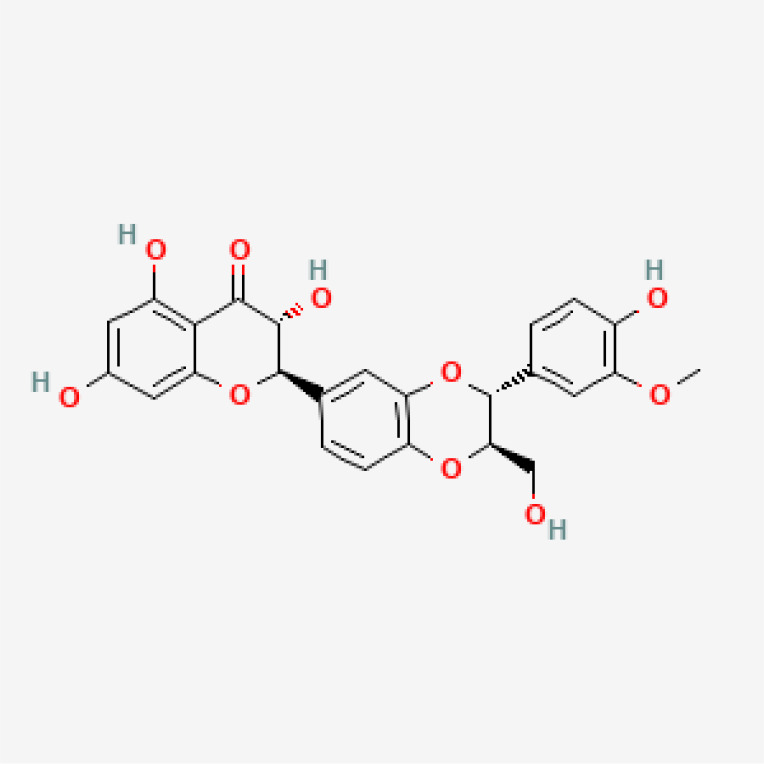	Myocardial I/R injury	Inhibit NF-κB pathway	(88)
2-methoxycinnamaldehyde	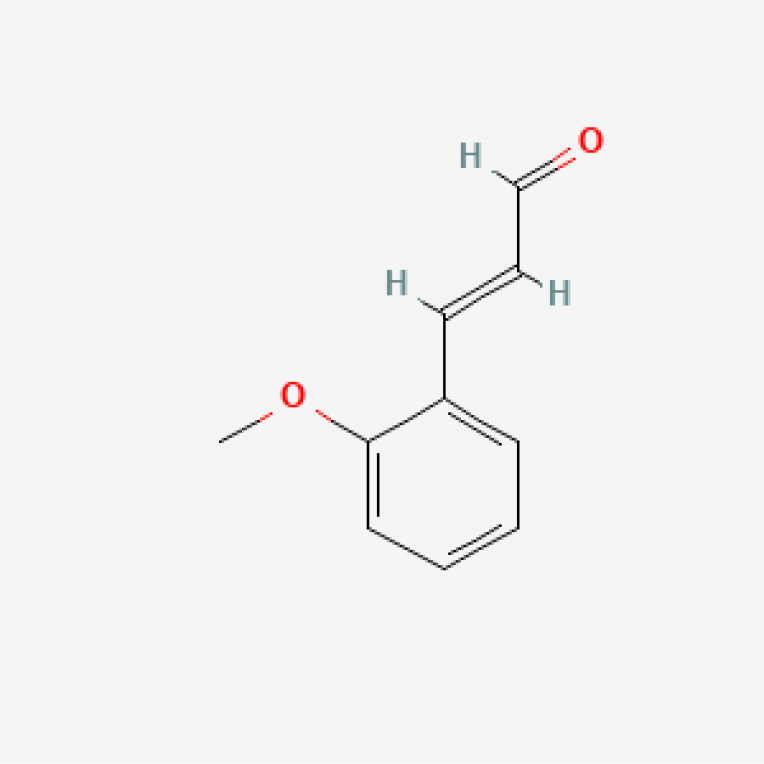	Myocardial I/R injury	Inhibit HO-1	(89)
Celastrol	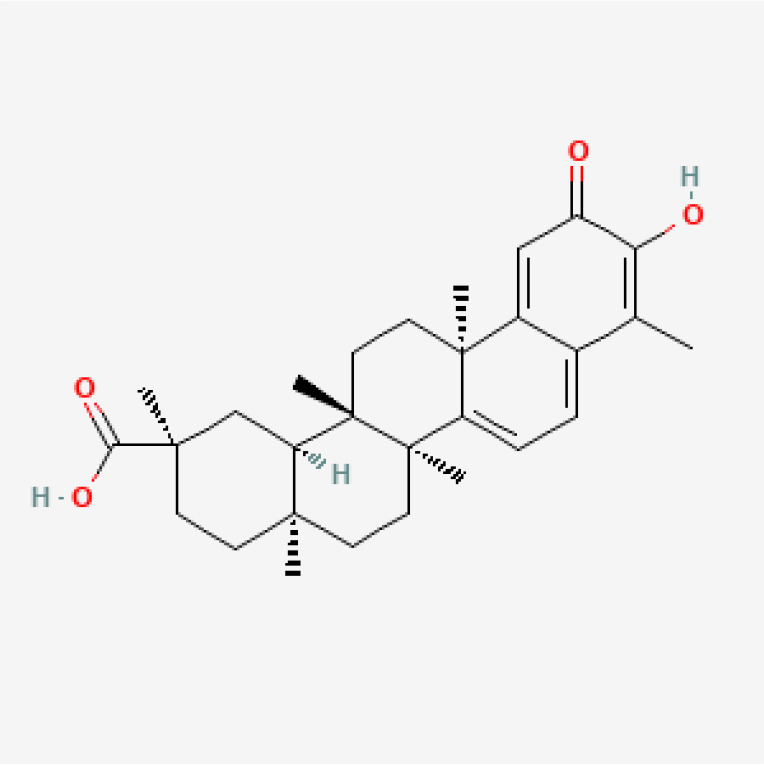	MI	Inhibit NLRP3 inflammasomes	(90)

TLR, toll-like receptor; I/R, ischemia reperfusion; MI, myocardial infarction; fMLP, formyl-methionyl-leucyl-phenylalanine;JAK, janus kinase; STAT, signal transducer and activator of transcription; NF-κB, nuclear factor kappa-B; HO, Heme Oxygenase.

## 6 Concluding remarks and Perspectives

Vast efforts have been continuously engaged over the past decades in the search for cardioprotective pharmacological agents in preventing immunoinflammatory response. Neutrophil recruitment is an important part of the immunoinflammatory response, which acts as a double-edged sword in the pathogenesis, progression, and healing of IHD. In the early stages of cardiac ischemia, the moderate inflammatory response caused by neutrophil recruitment lays the foundation for subsequent healing and repair by clearing dead myocardium and stromal debris. However, the occurrence of NR can promote the release of various inflammatory and chemotactic mediators, leading to cell damage and aggravating inflammatory response to myocardial injury. Therefore, how to inhibit the specific parts of the innate immune system that cause damage without affecting the healing process of ischemic myocardium and infarcted myocardium has become the research frontier of intervention in ischemic heart disease from the perspective of neutrophil recruitment. This review mainly discusses and summarizes the biological information of NR in the context of myocardial ischemia, which will contribute to a better understanding of their effects in the pathophysiology of IHD, and provide new avenues and methods for the prevention and treatment of IHD.

## Author contributions

YW: Writing – original draft. XS: Funding acquisition, Methodology, Writing – original draft. YWu: Methodology, Writing – review & editing. DL: Funding acquisition, Writing – review & editing.
